# Supplementary figures and images for: Endocrine modulation of primary chemosensory neurons regulates Drosophila courtship behavior
Source: PLoS Genet. 2022 Aug 23;18(8):e1010357. doi: 10.1371/journal.pgen.1010357 (PMC9439213; doi:10.1371/journal.pgen.1010357)

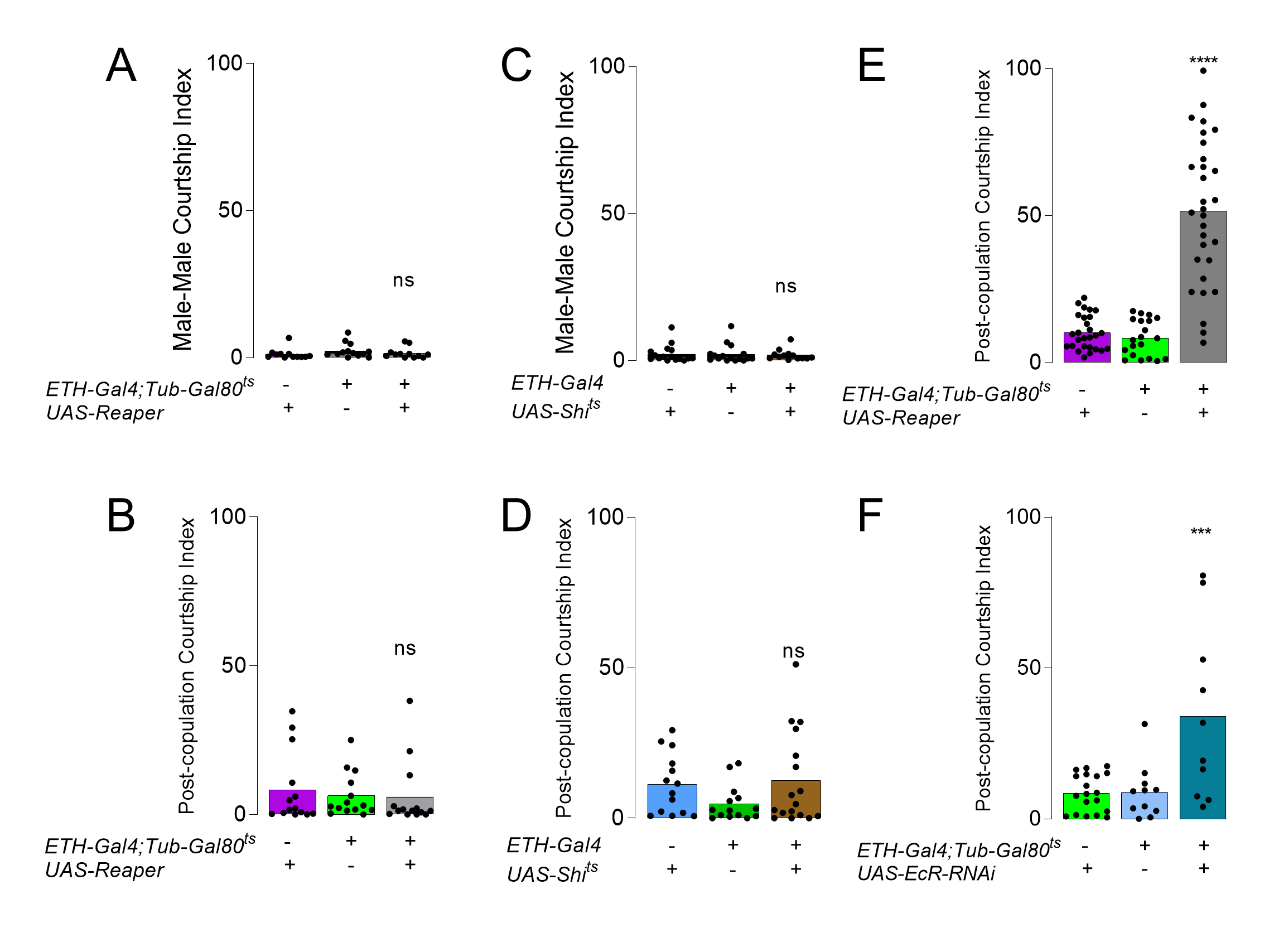

Supplement: S1 Fig — (A-D) Cold controls for male-male (A, C) and male-female (B, D) courtship indices (time spent courting over 600s) toward a wt counterpart for Inka cell-ablated (ETH-Gal4;Tubulin-Gal80ts>UAS-Reaper, A-B), Inka cell secretion-blocked (ETH-Gal4>UAS-Shits, C-D), and genetic controls kept in 18°C after eclosion (ANOVA, n = 15–20). (E-F) Post-mating courtship index (time spent courting over total time starting at dismounting, 600s) toward a wt female for Inka cell-ablated and genetic control males (E, ANOVA, n = 30) and males with EcR knocked down in Inka cells and genetic controls (F, ANOVA, n = 10–20). ns p>0.05; *** p < 0.001; **** p < .0001. (TIF) [file pgen.1010357.s001.tif]

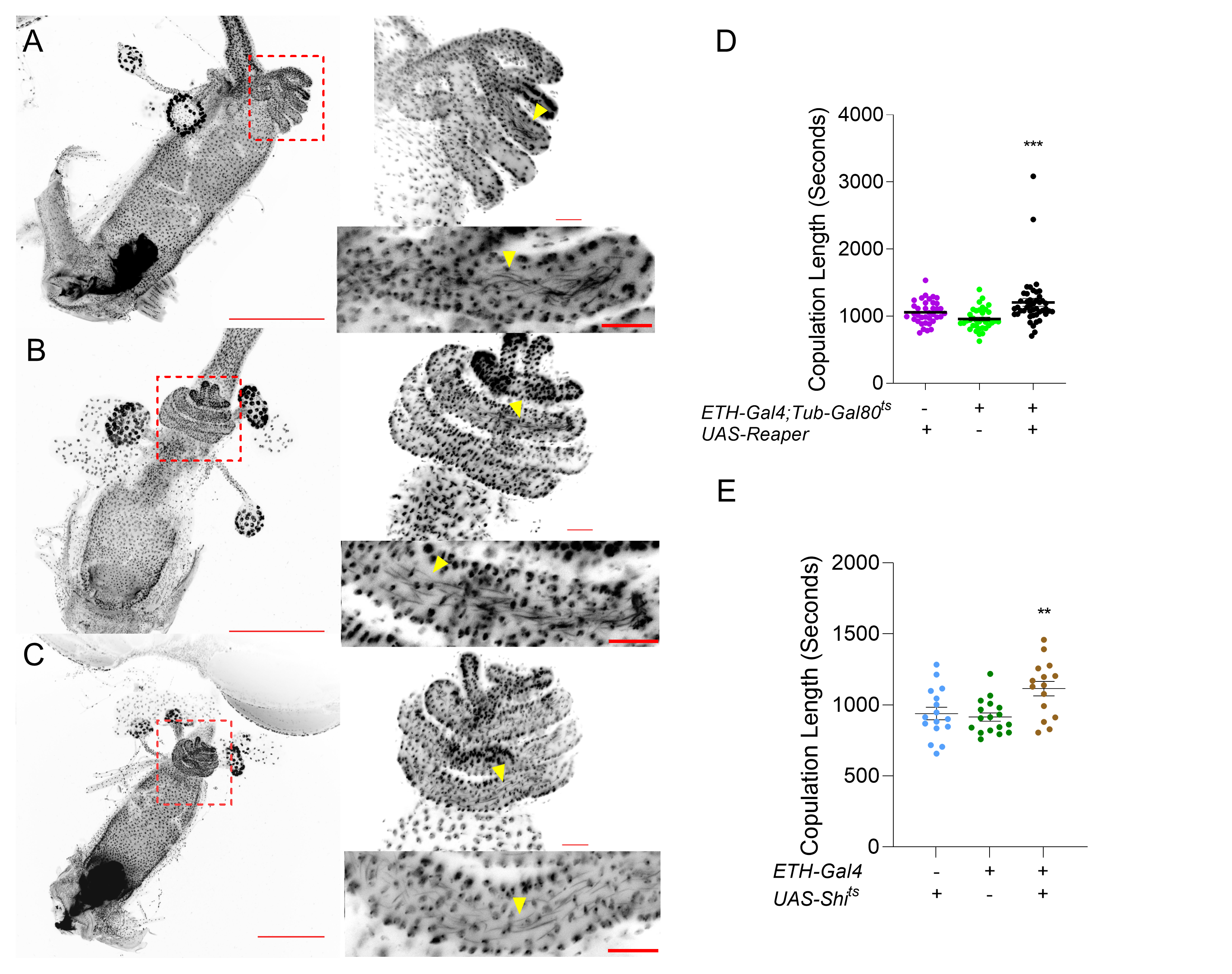

Supplement: S2 Fig — (A-C) DAPI-stained female reproductive tracts (Left, scale bar = 250 μm) from wild-type females, flash frozen 2 hours after mating to ETH-Gal4;Tubulin-Gal80ts/+ (A), UAS-Reaper/+ (B), or ETH-Gal4;Tubulin-Gal80ts/UAS-Reaper (C). Rod-like sperm heads are visible in the spermathecae (right, indicated by yellow arrowheads, scale bar = 25 μm). (D-E) Copulation duration (seconds from mounting to dismount) for Inka cell-ablated (ETH-Gal4;Tubulin-Gal80ts>UAS-Reaper)(A, ANOVA, n = 30–40) and Inka cell-blocked (ETH-Gal4>UAS-Shits)(B, ANOVA, n = 15–20). ns p>0.05; ** p < .01; *** p < 0.001. (TIF) [file pgen.1010357.s002.tif]

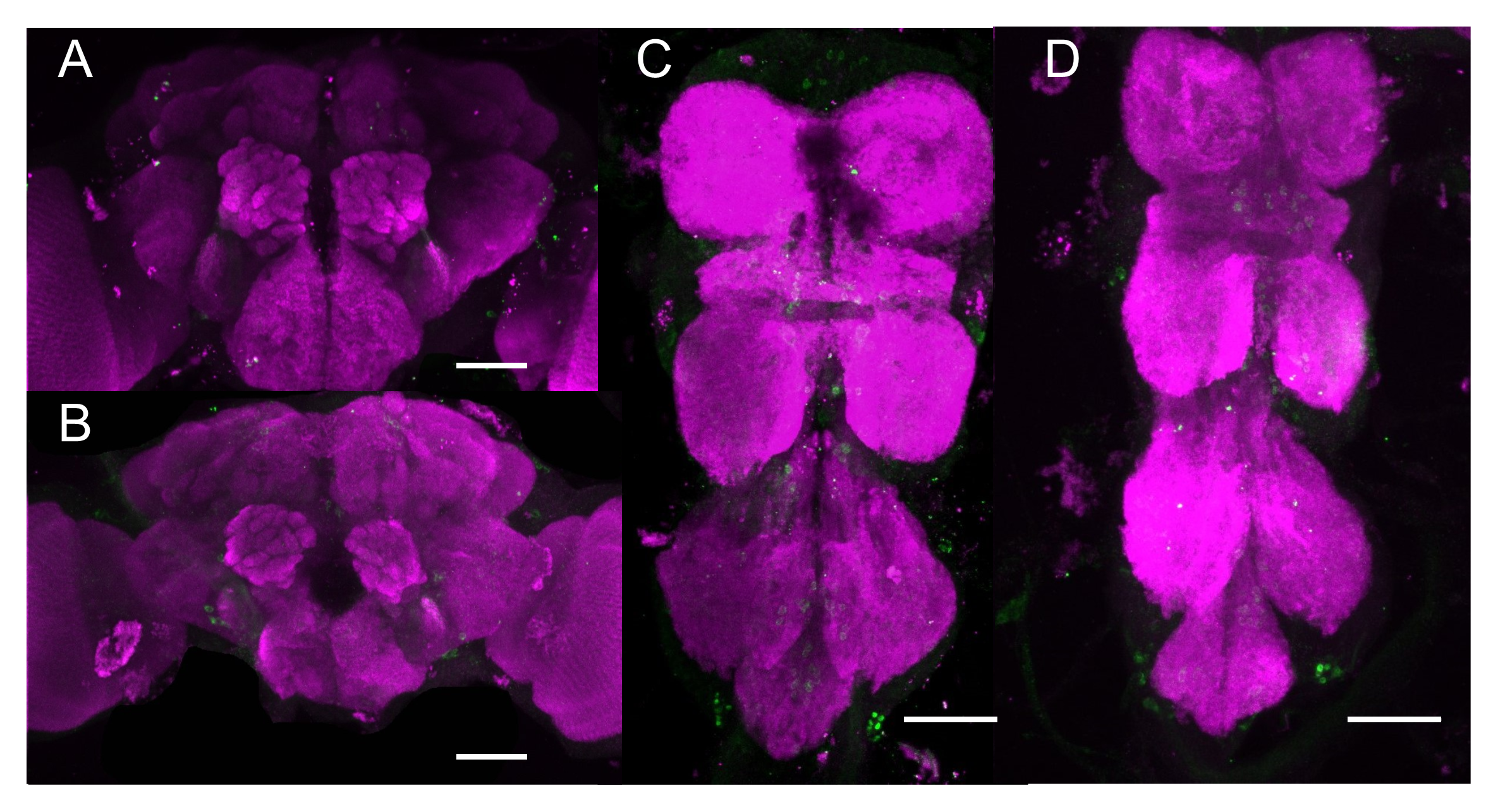

Supplement: S3 Fig — (A-B) Example confocal images of brains (A-B) and thoracic ganglia (C-D) from male (A, C) and female (B, D) flies of genotype UAS-mCD8>ETH-Gal4 (anti-GFP is green, anti-bruchpilot is magenta, scale bars = 50 μm). (TIF) [file pgen.1010357.s003.tif]

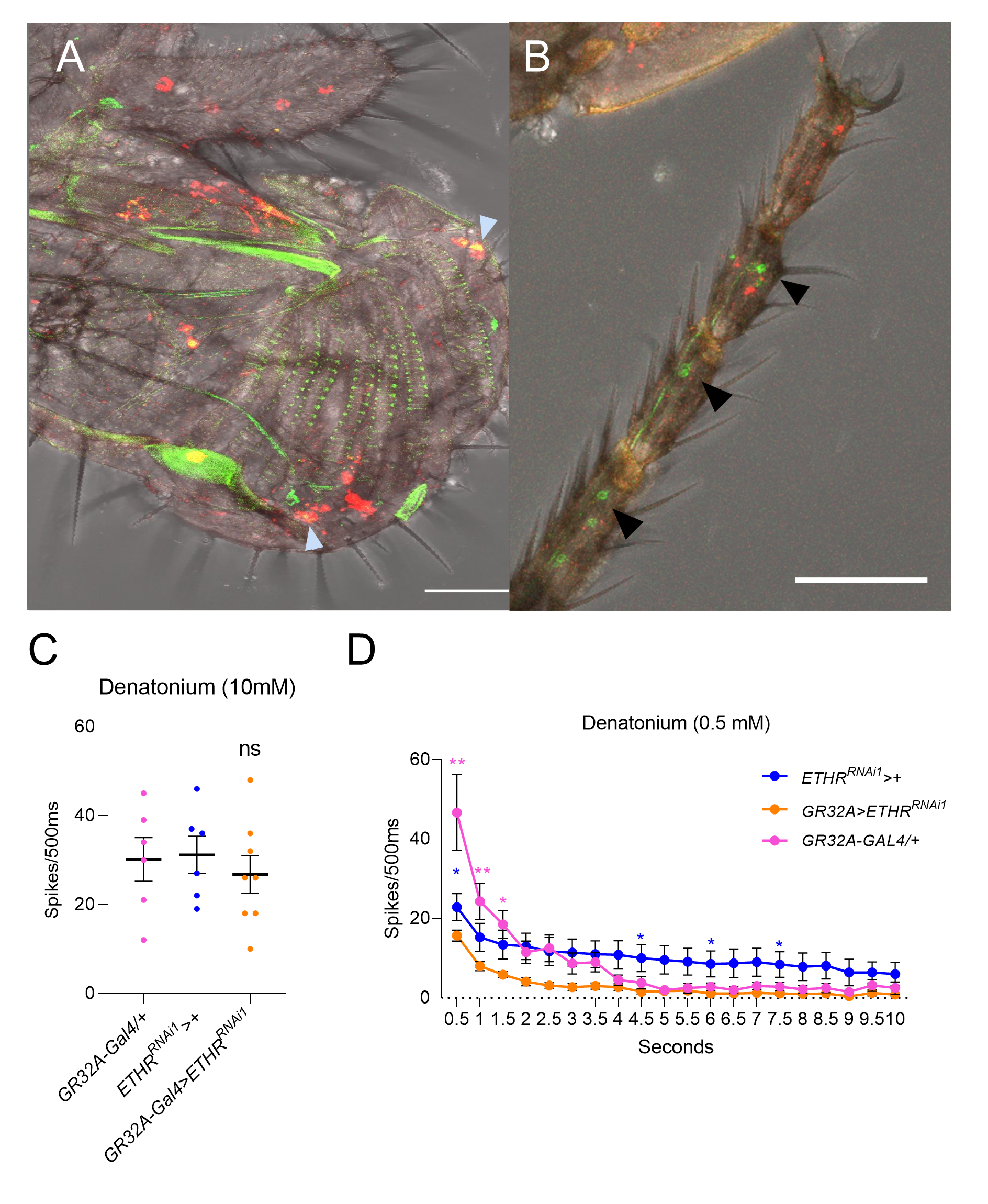

Supplement: S4 Fig — (A-B) UAS-GFP;ETHR-Gal4>AOP-mCherry;GR32A-LexA overlapping expression in labella (A, blue arrowheads) non-overlapping expression in tarsi (B, black arrowheads). (C) Mean spike response from extracellular recordings of S6 taste sensilla of GR32A-Gal4>UAS-ETHRRNAi1 and control males to 10 mM denatonium for 500 ms after contact (One-way ANOVA with Tukey’s test for multiple comparison, n = 6–8). (D) Spikes/500ms for each 500ms interval between 0 and 10 seconds after contact with 0.5 mM denatonium for GR32A-Gal4>UAS-ETHR-RNAi and genetic controls (One-way ANOVA followed by Tukey’s test for multiple comparisons, n = 6–7, color-matched asterisks indicate groups statistically distinct from test flies). (TIF) [file pgen.1010357.s004.tif]
