# Supplementary material for: Endocrine modulation of primary chemosensory neurons regulates Drosophila courtship behavior
Source: PLoS Genet. 2022 Aug 23;18(8):e1010357. doi: 10.1371/journal.pgen.1010357 (PMC9439213; doi:10.1371/journal.pgen.1010357)
Supplement: Attachment — Submitted filename: Reviewer responses.docx [file pgen.1010357.s005.docx]

Reviewer #1: The manuscript submitted by Meiselman et al is an important addition to the field of Drosophila courtship and endocrinology. The authors carefully characterize the role of the peptide hormone, ETH, in regulating male courtship behavior. Using the Gal4/UAS system to deplete ETH release or ETH receptor activity in certain sensory neurons, the authors link ETH activity to the regulation of male-male courtship and post-copulation courtship toward females. The authors also provide evidence that ETH regulates male courtship indirectly via Juvenile Hormone release from the corpora allata. The authors submit an interesting model whereby ETH influences male courtship through multiple pathways. I recommend this work for publication in PLOS Genetics with the minor revisions enumerated below.

*We thank the reviewer for their positive appraisal of our manuscript.*

- line 55: cues

*Fixed.*

- line 68–69: sentence clarification: As it reads, it's not clear if the authors mean that the JH analog rescues the ETH or JH deficiency. Pls specify.

*While ETH-deficient males showed no decline courtship in intensity after mating, treatment of ETH-deficient males with juvenile hormone analog restored (“rescued”) the decline. “Rescue,” as written, describes re-establishment of the decline in courtship intensity typically seen in wild-type males. We edited the line for clarification (Line 71-73):* “Juvenile hormone (JH) deficiency also relieves PCCI; treatment of ETH-deficient males with the juvenile hormone analog methoprene (JHA) reestablishes a decline in courtship intensity after mating.”

- line 76: What's an "allatotropin"? Pls define it in the text for readers less familiar with insect endocrinology.

*To avoid jargon, we replaced “*allatotropin*” with “*which stimulates synthesis and release of JH*” and added relevant citations (Line 79).*

- line 78: Readers unfamiliar with the genetic tools that target Inka cells will be curious to know how "clean" the ETH-Gal4 is. Pls clarify. How specific is the ETH-Gal4 to Inka cells in the adult? E.g., can the authors at least rule out CNS expression? In lieu of doing IHC, perhaps the ETH-Gal4 was carefully characterized in another paper; the authors could reference that paper and provide a clear statement about the specificity of the Gal4. I would recommend the same for all Gal4 drivers used in behavioral experiments of the paper. Ideally, the authors would provide adult CNS images for all drivers used in the paper.

*We have thus far utilized this ETH-Gal4 line in several papers (Lee et al., 2017, Meiselman et al., 2018, Lee et al., 2019) but have not investigated possible CNS expression. We therefor added a new supplementary figure (****S3 Fig****) which shows ETH-Gal4-driven expression of mCD8-GFP in male and female adults. As we’ve previously observed, there is almost no labeling in the CNS, with the exception of very faint labeling in the thoracic ganglion.Our arguments for ETH’s role in courtship rely not only on this data, but also ETH injection and knockdown of ETHR causing corroborating impacts on courtship. Whether these very weakly-labeled cells are relevant drivers of circulating ETH is beyond the scope of this paper.*

- line 86: An ETH rescue experiment would strengthen this section a lot. I recommend that the authors attempt to rescue the phenotype of ETH deficient males by injecting ETH.

*This is a thoughtful suggestion. We have attempted this in the past. After injection with saline or ETH copulation success declines dramatically during the hour after injection. We attribute this not only to the physical trauma of injection, but also the prolonged anesthetization necessary to carefully inject a group. Males can fully recover after 24 hours, and mate, but we expect injected, circulating ETH will be cleared from the hemolymph at this point, as ETH levels decline within the first hour following ecdysis (Zitnan et al., 1999).*

- line 92: Do the authors mean Fig. 1F? Also, the authors may consider combining this paragraph with the previous one, as it appears to be a continuation of the remarks re Fig. 1F-G.

*Excellent catch, fixed.*

- line 118: What is "ETHR-trojan-Gal4" and how exactly does it differ form "ETHR-Gal4" written below? Pls clarify and define in the text.

*Eliminated “Trojan” for consistency and added a citation (Diao et al., 2015) for readers interested in the line’s character and synthesis (Line 128).*

- line 119: From my understanding of the sentence, the authors are saying that the ETH-trojan-Gal4 labels interneurons that innervate the noted glomeruli, and are post-synpatic to the noted sensory neurons. They claim that these interneurons are also fru positive. Do the authors provide any evidence for that? Pls clarify or provide the data.

*The ETHR-Gal4 driver labels neurons that innervate several glomeruli, including VA1lm and DA1. These glomeruli are postsynaptic targets of sensory neurons that express OR47B and OR67D olfactory receptors, respectively. Fru^M^ is expressed in both of these sensory neurons and the glomeruli that they target (Stockinger 2005, Manoli et al 2005, Fishilevich et al 2005, Grosjean et al., 2011). We added these references and changed the text (Line 137-140) to read:* “…we identified neurons innervating VA1lm, and DA1 glomeruli, which are postsynaptic targets of olfactory sensory neurons which express OR47B and OR67D, respectively (Fig 3B, D) [34]. These sensory neuron subsets are *Fru^M^*-positive olfactory neurons which convey pheromone information to the brain to tune courtship [35, 36].”

- line 121: In Fig. 3A, I recommend including an illustration of a fly brain with the region shown in 3A boxed. At first blush, it's unclear what part of the brain we're looking at in 3A.

*We have transformed Figure 3 to an anatomy figure, and included not only higher quality pictures of the antennal lobes from both sexes, but the brains from which the antennal lobes are from to provide context.*

- line 135: The authors often say "ETHR-silenced" when referrning to RNAi-mediated depletion. To be more accurate and clear, I recommend "ETHR-depleted" or "ETHR-knockdown".

*The reviewer is correct that knockdown more accurately describes our manipulations. We have edited the text to remove all “silencing” references.*

- line 147: Why did the authors chose to test Gr32A function with a Gr33A mutant? To my knowledge, loss-of-function alleles of Gr32A are available. It would make more sense to use Gr32A mutants. Alternatively, perhaps the authors could use an RNAi against Gr32A.

*We did not have access to a GR32A null mutant. The current molecular model of bitter compound detection is that GR33A can form a hetero-oligomer with GR32A to sense bitter compounds, but certain ligands depend on either one molecule or the other (Dweck and Carlson, 2020) we hypothesized GR33A mutants might be rendered unable to utilize GR32A, but our new data suggests this is not the case. To directly assess the role of GR32A, we used the minos-mediated integration cassette (mimic) collection to eliminate GR32A in neurons (Figure 5A-B). We found GR32A-deficient males exhibit elevated male-male courtship behavior and post-copulation courtship, in line with the rest of our data. We felt this tangent was outside of the already very broad scope of the study, so we have removed the data from the manuscript.*

- line 151: It is indeed surprising that knockdown of ETHR in Gr32A-expressing neurons produced a PCCI phenotype, whereas loss of Gr33A (which should affect the function of Gr32A-expressing neurons) had no apparent effect. Can the authors speculate why this may have happened? Perhaps use of a Gr32A mutant would give different results.

*The reviewer’s prediction was, indeed, prescient. See above.*

- line 162: The authors should mention was "CA" is.

*“CA” refers to the juvenile hormone-producing gland the corpus allatum, however, we did not add a clarifying indication of the abbreviation. We added corpus allatum (CA) to line 26 for clarification.*

- line 166–168: This sentence is confusing. The authors write, "restored the normal postmating decline of courtship," but then go onto to say that JH is "insufficient to completely account for elevated post-copulation courtship in ETH-deficient males." As I see it (and what I think the authors are saying), giving JH to ETH-deficient males partially restored the postmating decline of courtship, which suggests that ETH exerts its effects through JH but also directly upon the sensory system.

*The reviewer’s assumption is correct. We’ve corrected (Line 191-193) to* “Methoprene treated, ETH-deficient males significantly decreased courtship after mating, but courtship after mating was still elevated compared to controls (Fig 5F).”

Along these lines, the authors should state how much methoprene they gave the males in the legend of Fig 4D. Additionally, it is not obvious how we can rule out the possibility that the partial rescue was due to insufficient amounts of methoprene. What if administering >300 nM better rescued the behavior of the ETH-depleted males, possibly even to full levels?

*We agree that dosage should be immediately obvious in the figure and have updated Fig. 5E to reflect the dose of 5 pg/male, a saturating dose, which was the same dose used on males to completely rescue JH-deficiency-induced declines in male reproductive capacity (Meiselman 2017), and courtship memory (Lee 2021).*

- line 169: In Fig 4D, I recommend that the authors include a wild-type control so the partial rescue can be more apparent to the reader.

*We added wild-type courtship index before and after copulation to Fig. 5E, which we hope offers clear context for the degree of rescue by methoprene treatment.*

- line 403: missing (D)

*Thank you for the catch. Updated.*

Reviewer #2: In this interesting paper the authors describe significant new roles for the Ecdysis-Triggering Hormone (ETH). They observe three aspects of courtship that are affected by reduction of ETH or silencing/ablation of cells that produce ETH:
- Injected ETH reduces male-female courtship.
- Ablation of INKA cells, where ETH is produced, increases male-male courtship.
- Silencing of the same cells abolishes post-copulation courtship inhibition.
These deficiencies can be mapped to reduced ETH action in a subset of sensory neurons, and in one case to lower sensitivity to stimuli.

The most significant and novel of these observations is the effect of reduced ETH action on Post-copulatory Courtship Inhibition (PCCI). Mutant males don’t suppress courtship after successful copulation. Based on their examination where ETHR is present, the authors test the roles of several candidate cells. They find that ETHR is required in Gr32 expressing neurons in the labellum and in OR76D antennal glomeruli.

These are really interesting new aspects of the complex physiological adult role of ETH and demonstrate how multi-faceted the adult roles of endocrine regulators can be. The experiments are carefully crafted and done and support the authors’ interpretations well.
I have a few questions and suggestions:

*We thank the reviewer for their positive appraisal.*

Figure 1:
1. I am a little surprised that mature control animals would only show a 50% copulation rate. Did the authors optimize their assay (and if so how) for this outcome, which would allow them to actually observe an enhancement of copulation success that might otherwise be hidden by a “ceiling effect”?

*We conduct experiments at 22 degrees Celsius rather than the commonly used 25°C, which may contribute to our relatively lower mating rate. We selected these conditions for consistency with our previous courtship investigations, and agree the lower intensity of courtship relative to other published results does accent the increased courtship observed as a consequence of our manipulations.*

Figure 2:
The SWITCH experiment is a nice experiments to address the question whether the defect lies in receiver or sender.
1. Have the authors tested a SWITCH constellation where both the first and the second mate are ETH deficient?

*We have not collected a complete dataset, but have anecdotally tested this with 6 males. As the reviewer might expect, ETH-deficient males still courted females intensely. ETH-deficient males court far more than controls in every condition we’ve tried.*

Figure 3:
1. The authors show the expression of ETHR-Trojan-Gal4 in distinct antennal lobe neurons. Figure A is not well suited to identify the relevant glomeruli. Without double staining, or at the very least images of the separate channels, outlining and naming the glomeruli (perhaps including a schematic) this is not clear.

*In response to reviewer suggestions, we have included an extra anatomy figure highlighting the sexually dimorphic expression of ETHR and clearly delineated all 5 glomeruli innervated by labeled olfactory sensory neurons.*

2. I believe it would be a good idea to include the S2 fig images in Figure 3.

*We have reorganized the figures, adding more images of male and female periphery (Fig.3G-J).*

3. The authors’ finding that Gr32 neurons with ETHR knockdown have reduced sensitivity is intriguing. However, it is not zero. Does the response increase with prolonged exposure or with higher concentration?

*We attempted both prolonged exposure and a higher concentration of denatonium during revision. We tested response of GR32A-Gal4>UAS-ETHR-RNAi and genetic controls to 10 mM denatonium (below) and, as was the case with 5 mM ETHR-RNAi had a lower spike average than controls, but it was not significant. We also observed spike frequency during each 500 ms interval over 10 seconds after denatonium contact in GR32A-Gal4>UAS-ETHR-RNAi and genetic controls. GR32A>ETHR-RNAi mean spikes reach baseline slightly before its genetic controls. These experiments were conducted on a different rig on flies reared on different food then the previous data, so the data will not be combined with the previously collected data and thus added to Figure S4 C-D.*

4. Why did the authors use Denatonium in their recordings? Why not 7T?

*We did not believe it would be possible to dissolve the non-polar 7-Tricosene in TCC solution, which is necessary for recording from taste sensilla. We nonetheless attempted to get it in and found vigorous vortexing was sufficient to mix in 7T. As shown in the dose-response curve (Fig. 4D), GR32A-Gal4>UAS-ETHR-RNAi males showed a reduced response to high concentrations of 7-Tricosene.*

Figure 4:
1.Why was GR33-/- chosen and not GR32-/-?

See responses above. *GR33A* mutants have been replaced with *GR32A* knockdown.

2. The fact that Gr33-/- did not show a phenotype, but GR32A-Gal4-UAS-ETHR RNAi has not been discussed

*See above.*

3. I think the role of JH deserves a little deeper discussion to describe its roles in different contexts. It appears to be a hormone that is important to “get it right” for wildtype courtship: Normal male-female courtship (enhance courtship when needed), normal postmating inhibition (suppress courtship when it would be inappropriate), perhaps by enabling correct recognition of pheromonal and other cues.

*We agree with this sentiment, and have added the following sentence to the ETH-JH signaling portion of the discussion to emphasize courtship’s dependence upon this critical gonadotropin (Line 247):* “Timing of circulating JH appears to be a critical for the courtship status quo, with too much or too little disrupting proper courtship behavior. In addition to its modulation of perception,…”

Additonal comments/questions:
1. The authors have previously shown that lower ETH results in reduced courtship memory, but only mention it in passing in the discussion. Are the mutant males described here capable of forming a courtship memory? I would like to see a deeper discussion how reduced courtship memory might play into the current findings.

*Courtship memory is defined in these past studies as reduction of male courtship activity following a conditioning protocol where the male is paired for a given period of time (courtship conditioning) with a mated female. Following courtship conditioning, the male is paired with a virgin female and reduced courtship is observed relative to control males. This reduction in courtship is attributed to previous rejection by the mated female. In this study, males presumably have a memory of successful courtship instead of rejection and failure. How this memory would modulate post-mating courtship index has not been investigated in this study. We have added a statement to the Discussion in this regard.*

2. The authors mention that it is unlikely that the absence of PCCI is caused by reduced mating time, in fact it appears slightly longer. Do the mutant males transfer sperm?

*We have shown in a previous work that ETH-deficient males can sire progeny, albeit at a reduced level (Meiselman et al., 2017), but we agree this point is worth illustrating. We have included this point in the text (Line 99) and updated S2 Fig to be devoted to this point, now including images of spermathecae from females recently mated to ETH-deficient (ETH-Gal4;TubGal80^ts^>UAS-Reaper) males with DAPI staining and sperm heads pointed out.*

Reviewer #3: In their manuscript “Endocrine Modulation of Primary Chemosensory Neurons Regulates Drosophila Courtship Behavior”, Meiselman et al. study the role of Ecdysis Triggering Hormone (ETH) in modulating courtship motivation. For this they look at postcopulatory courtship inhibition (PCCI), the phenomenon of strongly reduced courtship activity of male Drosophila shortly after a copulation. They find that ETH is required for PCCI and the suppression of male-male courtship. Based on a “mate and switch” experiment, they propose that the effect is due to an internal state change of the male, i.e. reduced male detection of courtship inhibitory cues on recently mated females and other males. The authors show that ETH receptor is required in chemosensory neurons Gr32a positive of the labellum for their full sensitivity and inhibition of male-male courtship. ETH receptor knockdown in Gr32a or Or67d sensory neurons also leads to abolishment of the PCCI effect, suggesting that ETH signaling is required for the function of these neurons in sensing inhibitory courtship cues.

The paper addresses an important topic, the neuronal and genetic basis of motivational control of reproductive behaviors. The results are interesting, but main mechanistic questions remain unanswered. Not all results are presented in a clear way and the manuscript and data could be organized in a better way. The Method part is insufficient, not all important experimental detail is explained. Some of the experiments lack critical controls that would allow for an overall more careful, comprehensive and insightful interpretation.

*We thank the reviewer for this insight. We have elaborated on experimental detail in the Methods section, particularly the courtship assays. Which we believe addresses the criticism.*

Major points:
1) Inka cell ablation/secretion block: can the authors exclude that killing of Inka cells has other effects than reduction of ETH levels? Do Inka cells possibly secrete other compounds than ETH? Can they specifically affect ETH synthesis in adult Inka cells and confirm their results? The authors inconsistently use “Inka cell ablated” and “ETH deficient” males for denoting the same experimental animals, which is confusing.

*Our studies over the past 25+ years indicate that ETH and related peptides expressed by the ETH gene are by far the predominant secretory products of Inka cells, which are quite specialized for ecdysis regulation prior to adulthood. Furthermore, expression of the ETH gene is dependent upon ecdysone receptor activation. Upon knockdown of ecdysone receptor expression in Inka cells in Figure S1F, we found relief of post-copulation courtship inhibition. This finding complements our findings under conditions of ETH-deficiency and knockdown of ETHR in GR32A neurons and are consistent our overall conclusion that compromise of ETH signaling, both from the viewpoints of ligand deficiency and receptor knockdown, leads to the PCCI phenotype.*

2) In Figure 1, temperature controls for the temperature dependent tub-Gal80ts experiments and the Shits experiments are missing, the data is not thus not conclusive.

*We have included cold controls (males kept at 18°C for the first 4 days, no exposure to the permissive 29°C) for male-male courtship and male-female post-copulation courtship in Inka cell blocking/killing experiments in Supplementary Figure 1A-D.*

3) The authors use two assays to assess courtship inhibition toward inappropriate targets: courtship toward mated females (PCCI) and toward males. I am missing a clear discussion how they think these assays differ. Do the authors think that they are testing the same with the two assays, i.e. response to inhibitory courtship cues cVA and 7-T, but PCCI is a more sensitive readout? In the manuscript, differences/commonalities between the assays is not made clear. Thinking a bit more, it is maybe not “surprisingly” (line 151) that PCCI would required both Gr32A and Or67D neurons, whereas male male courtship inhibition is more robust and still in place when only Gr32a is affected.
Fig3D and Fig4A,B belong, in my opinion together and could maybe presented in one figure, also in a more consistent way? For example, why are 2 RNAi lines only used for PCCI, and why is Or47b-Gal4 only tested for male male courtship?

*We agree with the author’s perspective. There are two key differences that make male-male courtship and post-copulation courtship distinct: 1. Males and mated females have different appearances, offer different types of behavioral feedback when inappropriately courted (such as female ovipositor extrusion), and have different pheromonal profiles. Males will court a slowly moving magnet, so it is not completely obvious what degree each of these characteristics have on the ultimate decision to court, but we see clear differences here, especially with OR67D, suggesting there are critical components which may be under the control of ETH. 2. When males mate, they change state. They form a memory of that mating which influences future courtship decision-making, and their courtship drive declines.*

*We have added the following passage (Line 65):* “Inappropriate courtship toward males and females after mating suggests ETH-deficiency obviates not only the variety of courtship-inhibiting olfactory and gustatory cues that distinguish each [14-16], but also any changes in memory or internal state that result from successful copulation [17].” *in the introduction so readers understand the insight provided by each assay.*

*We have removed “surprisingly” to avoid editorializing.*

4) Inconsistency of presenting PCCI data in Fig1F and Fig4D versus Fig4A-C. Why is the pre-copulation data not shown in Fig4A-C? Presentation as paired data seems more conclusive to me.

*We appreciate the reviewer’s suggestion, but we do not think adding precopulation courtship index for any genotypes other than ETH-Gal4;TubulinGal80ts>UAS-RPR will add valuable information to the story. This choice of data presentation was made because Inka cell ablation eliminates the decline in courtship intensity typically observed after mating. This was not the case for any other genetic manipulation, including manipulations in 4A-C. We posit here that this is because ETH has at least three downstream targets that contribute to post-copulation courtship inhibition. Elimination of each, alone, does not eliminate the decline observed after mating. As premating courtship index is typically highly variable due to the differing lengths of time to copulation, we find it redundant and not informative to report pre-copulation courtship in each of these cases. We therefore chose to report comparisons between the total amount of post copulation courtship observed in each genetic manipulation rather than including pre-copulation data to be concise.*

5) Fig 2: When is ETH signaling required in the sensory neurons, during development or in the adult? Does manipulation of ETH signaling for example change sensory neuron survival, wiring/morphology? There are many ways to restrict RNAi knockdown of ETH receptor temporally and by this explore the mechanism of changed response to inhibitory cues.

*Several pieces of evidence support the idea that neural dependence on ETH is an adult-specific phenomenon. First, we show in Figure 1 that suppression of Inka cell release specifically in the adult stage de-represses male courtship, and injection of ETH (Figure 1C) causes acute (within 1 hr) suppression of male courtship. We show in Figure 2A-B that this deficiency is a consequence of pheromone perception impairment. In Deshpande et al. (2019), we knocked down ETHR specifically in the adult stage using pan-neuronal ETHR knockdown (elav-Gal4;Tub-Gal80^ts^/UAS-ETHR-RNAi), reared at restrictive temperature (18C) until eclosion and then moved to permissive temperature (29C) and assessed male-male courtship on day 4. Male courtship was elevated even when knockdown was temporally restricted to the adult stage. Using the same flies, we have examined post-copulation courtship index and found their courtship after copulation was disinhibited at levels similar to or above those of males with pan-neuronal ETHR knockdown through their development (Added to figure S1F). Finally, we show changes in action potential responses within GR32a primary sensory neurons to ligands, arguing that ETHR silencing causes cell-autonomous changes in firing, rather than wiring. We believe these data support the model that circulating ETH in the adult stage tunes courtship behavior.*

6) The finding that ETHR knockdown alters the response profile of taste hairs is very interesting. It is however not entirely clear why this is demonstrated with denatonium when the relevant candidate substance is 7-T or extract from male cuticular hydrocarbons. Tests with actual inhibitory courtship cues would be more compelling here. Also, what are the datapoints in Fig3E and 3G representing: tested hairs, animals, repeated stimulus presentations?

*We were able to successfully execute this experiment (Fig. 4D). Each represents an animal that was stimulated only once, and we have updated the Methods to make this clear.*

7) The last section of the results, line 155 following (Juvenile Hormone regulates post-copulation courtship) is the least clear part of the manuscript. The authors perform methoprene treatment of Inka cell ablated males and find that this partially restores PCCI. However, it is not apparent how JH or its analog methoprene exerts this effect and if sensory neurons are involved. To make the mechanistic link with their previous findings, test their model presented in Figure 4E and disambiguate central motivation circuits from sensory neuron function effects, it would be good to test methoprene treatment in Gr32a and Or67d mutants and flies with ETHR knockdown in sensory neurons.

*This work is complemented by several excellent works conducted in Michael Crickmore’s lab, on the role of juvenile hormone in mating satiety and courtship drive. As displayed in the model in* ***Figure 5E*** *we suggest that JH acts directly on the brain, more specifically, courtship-reporting neurons which affect the FruP1 hub regulating mating drive (Zhang et al., 2018). We have nonetheless attempted to methoprene rescue the OR67D mutant since JH regulates olfactory courtship neurons (i.e., OR47B neurons, HH Lin et al., 2016). As predicted by our model, methoprene treatment of mutants did not affect elevated PCCI. We have included the data below but have left it out of the manuscript as we feel the relationship between JH and the sensory neurons is beyond the scope of this paper.*

Response Figure 2 (**A**) PCCI for males homozygous or heterozygous mutant for *OR67D*, treated with either ethanol alone or 5 pg of methoprene in ethanol (n=12-16, ANOVA). ns p>0.05.

Minor points:
1) Abstract :«and critical for mate choice and courtship inhibition after the completion of copulation”- the formulation “critical for mate choice after completion of copulation” is a bit confusing/misleading. The term “postcopulatory mate choice” is often used in regards to female decisions about usage of received sperm and control of fertilization. I assume the authors mean the male has a lower courtship motivation right after one copulation and then “chooses” not to mate a second time right afterwards. Since they study courtship motivation rather than “mate choice” sensu stricto, I suggest changing wording here.

*We agree with the sentiment here, we have deleted “mate choice,” which we were using to refer to the aberrant male-male courtship, to simply emphasize the post-copulation courtship. It now reads “*Here, we show Ecdysis-Triggering Hormone (ETH) is a regulator of male courtship behavior in Drosophila melanogaster, and critical for courtship inhibition after the completion of copulation.*”*

2) Line 55: “queues”> cues??

*The reviewer is correct, “queues” is now replaced by “cues.” (Line 16, 17, 38, 55, 66, 101, 102)*

3) Line 140: “ETHR Silencing” > confusing terminology- the receptor is not “silenced”, but its translation diminished by RNAi or it is absent in the mutants.

*We agree with the reviewer and have replaced all mentions of “silencing” with “knockdown.”*

4) Figure 3A: red green color scheme is inconsiderate for color blind.

*We replaced the red and yellow with the more traditional green and magenta.*

5) Line 205: Reference 37 should be rather cited after the following sentence.

*Correct. We added the correct reference which discovered the calcium mobilizing properties of fly ETHR (Iversen et al., 2002, Park et al., 2003).*
